# Solid Organ Transplantation During the COVID-19 Pandemic

**DOI:** 10.3389/fimmu.2020.01392

**Published:** 2020-06-16

**Authors:** Hedong Zhang, Helong Dai, Xubiao Xie

**Affiliations:** ^1^Department of Kidney Transplantation, The Second Xiangya Hospital of Central South University, Changsha, China; ^2^Clinical Research Center for Organ Transplantation, Changsha, China; ^3^Clinical Immunology Center, Central South University, Changsha, China

**Keywords:** COVID-19, solid organ transplantation, immunosuppressant, transplant safety, clinical characteristics

## Abstract

Since December 2019, the ongoing coronavirus disease 2019 (COVID-19) pandemic has significantly affected solid organ transplantation (SOT) worldwide and has become a threat to the lives of SOT recipients. Here, we have reviewed, condensed, and organized the available information on COVID-19 to provide recommendations to transplant healthcare workers. Our review of reported cases shows that the symptoms of SOT patients with COVID-19 are similar to those of the normal population, but their severity and outcomes are worse. Thus far, there is no evidence that severe acute respiratory syndrome coronavirus 2 (SARS-CoV-2) directly causes permanent damage to kidney, liver, or heart allografts.

## Introduction

In December 2019, a cluster of patients with pneumonia of unknown cause were reported (1). The pathogen was promptly isolated on January 7, 2020 and its whole genome was shared later on January 12, which identified it as a novel member of the β coronavirus family (2, 3). On February 11, 2020 this virus was named severe acute respiratory syndrome coronavirus 2 (SARS-CoV-2) by the International Committee on Taxonomy of Viruses (ICTV) (4); on the same day the disease caused by SARS-CoV-2 was named as coronavirus disease (COVID-19) by the WHO (5). On March 11, 2020, the WHO made the assessment that COVID-19 can be characterized as a pandemic (6), and as of April 17, 2020, nearly 2,074,529 cases and 139,378 deaths have been confirmed worldwide (7).

Droplet and contact transmission are the main routes of viral transmission; airborne transmission is only possible in specific circumstances such as endotracheal intubation, bronchoscopy, and open suctioning (8, 9). Although SARS-CoV-2 has been cultured from the stool (10), fecal–oral transmission has not been reported. The general population lacks immunity and is susceptible to SARS-CoV-2 due to the novelty of the virus. The average incubation period for COVID-19 is 5.2 days (95% confidence interval [CI], 4.1–7.0) with the 95th percentile at 12.5 days. The basic reproduction number (R0) value has been estimated to be 2.2 (95% CI, 1.4–3.9) (11). It should be noted that asymptomatic patients with SARS-CoV-2 account for 1.2% of the infected cases according to a retrospective study of 72,314 confirmed cases in China (12). Other studies have concluded that the percentage of asymptomatic patients range from 8.6 to 15.7% (13). Further, evidence indicates that asymptomatic patients can also transmit the disease (14, 15).

The spread of COVID-19 has significantly restricted the transplantation program in epidemic areas due to the high risk of infection in immunosuppressed patients, risk of transmission in health workers, impact of travel bans on organ procurement organizations' (OPO) activities, and lack of medical resources in certain circumstances. In this paper, we explore ways to safely continue transplantation under the threat of COVID-19 and review the current reported cases of organ transplant in COVID-19 patients.

## Organ Transplantation Proceeds With Extra Precautions in Low-Risk Areas

### Transplant Activity Report During the COVID-19 Pandemic

In March 2020, organ transplant centers in Wuhan completely halted all transplant surgeries. A suspension on living donor transplants, pancreas transplants, and renal transplants was first considered in other less affected regions; however, lifesaving urgent transplant surgery still proceeded in most centers (16, 17). Statistics showed a drastic 90.6% and 51.1% reduction in deceased donor transplant in France and USA, respectively, which is mostly driven by kidney transplant (18). Total amount of all organ transplants reduced from 100~150 to 40 transplants per month in Netherlands (19). Kumar et al. have suggested a phase down approach to new transplant activity during this unprecedented crisis. The percentage of transplant activity reduction should be based on risk tolerance, the degree of local COVID-19 activity, and medical resource capacity (20). Generally, according to the transmission patterns defined by the World Health Organization (21), when sporadic cases and clusters of cases with clear traceable transmission chains are present, transplantation can be performed with precautions. However, in regions where community transmission (large number of untraceable cases) is on the rise, a temporary tiered suspension on transplantation is recommended due to increased amount of infection risk among patients and healthcare workers (22).

### Donor and Recipient Screening

No donor-derived COVID-19 case has been reported thus far. Although 10.4–15% of COVID-19 patients have been detected with serum SARS-CoV-2 nucleic acid (RNAaemia), no infectious live virus has been found in the blood (23, 24). Angiotensin-converting enzyme 2 (ACE2), which has been identified as a receptor for SARS-CoV-2 (25), is abundant in virtually all organs including the lung, kidney, liver, heart, and intestine (26); this implies that SARS-CoV-2 viremia could possibly infect any transplant organ and conceal itself until the immunosuppressed status exists. Postmortem autopsy has shown that SARS-CoV-2 mainly exists in the lungs (27, 28), although involvement of the kidney, liver, and heart has also been reported (29, 30). Considering the real risk of donor-derived infection under immunosuppression and the risk of viral transmission to health workers during coordination, procurement, surgery, and nursing, both donor and recipient screening should be mandated in the affected areas.

Due to the existence of asymptomatic patients, clinical screening methods such as inquiry into potential exposure and suspected symptoms, laboratory blood examination, or a chest computed tomography (CT) (31) are not sufficient to completely rule out the possibility of infection. A nucleic acid test (NAT) remains the cornerstone of COVID-19 screening. The positive rate using nasopharyngeal swabs (63%) is lower than that using the bronchoalveolar lavage fluid (93%) and the sputum (72%), but is significantly higher than using oropharyngeal swabs (32%); nasopharyngeal swabs have the highest viral load among all types of clinical specimens (32). Because of its accessibility and sensitivity, NAT nasopharyngeal swabs are preferred for screening purposes (33). Universal NAT testing should be recommended in community transmission areas (20, 22). However, due to high false negative rate of one-time NAT testing, if feasible, two consecutive NAT tests conducted ≥24 h apart should be considered (34, 35).

However, when life-saving urgent transplantation is required and NAT kits are limited, testing should be done in the following order of priority: suspected donor/recipient, donor, and recipient, because the donors have a higher exposure risk (ICU experience for instance) compared with low-risk recipients who follow social distancing rules. Suspected donor/recipient cases (different from the suspected cases for epidemiological surveillance purposes) are determined based on the following (36–38):

- Possible exposure to COVID-19 confirmed or suspected patients.
- Suspected symptoms such as fever (reported symptom prevalence: 83–99%), cough (59–82%), fatigue (44–70%), anorexia (40–84%), shortness of breath (31–40%), sputum production (28–33%), and myalgias (11–35%).
- Conditions such as lymphocytopenia (83.2%), thrombocytopenia (36.2%), and leukopenia (33.7%).
- Abnormal CT results (86.2%) with ground-glass opacity (56.4%), and bilateral patchy shadows (51.8%).

Any suspected donor/recipient (who fulfills any of the above conditions) should be screened by NAT for SARS COV-2. Any COVID-19 confirmed donor/recipient, suspected donor/recipient unable to rule out COVID-19, and donor/recipient with fever of unknown cause should be contraindicated for organ transplant.

### Safety of OPO Coordinators

In regions where organ transplant programs are still ongoing, OPO coordinators generally have to travel more compared with transplant doctors and nurses and social contact is unavoidable. It is thus essential to impart training on epidemiology, prevention, risk assessment of COVID-19, and psychological tutoring on stress coping (39). Coordinators should perform risk assessment depending on the epidemic situation of the area where the donor originates from, the hospital/ICU where the donor currently stays, wear personal protective equipment, and practice hand hygiene during the whole donation coordination. Further, they should reduce unnecessary in-person contact by utilizing online communication tools.

## COVID-19 in Transplant Recipients

According to a survey conducted on 88 major transplant centers in the United States between March 24 and March 31, 2020, 31 (35.2%) respondents reported 148 COVID-19 SOT patients altogether. Of these patients, 80 (54.1%) were mildly ill (no pneumonia), 31 (20.9%) were moderately ill (pneumonia), and 37 (25.0%) were critically ill (17). Although the classification is subjective, the results indicate a greater disease severity compared with normal COVID-19 patients. Herein, we reviewed all reported COVID-19 SOT cases prior to April 17, 2020 to show the manifestation, treatment, and prognosis.

### COVID-19 in Kidney Transplantation Recipients

Kidney transplantation (KT) recipients account for the largest proportion of SOT recipients (40), which explains why they form the majority of COVID-19 SOT patients (103/148, 69.6%) (17). As of April 17, 2020, 27 COVID-19 KT patients have been reported on PubMed. The mean age was 53.4 ± 16 years, and the median post-transplantation (post-TX) interval was 4 years [interquartile range (IQR): 1.9–9.6]. No pattern was observed between the KT recipients' susceptibility and post-TX interval. It has been suggested that the atypical symptoms and absence of fever seen in COVID-19 SOT patients might be due to immunosuppression (41–43). Our review (Table 1) shows that COVID-19 KT patients presented with fever (25/27, 92.6%), cough (20/27, 74.0%), dyspnea (12/27, 44.4%), myalgia (8/27, 29.6%), fatigue (7/27, 25.9%), and sputum production (4/27, 14.8%), which is similar to COVID-19 symptoms in the general population (37). The gastrointestinal symptoms seen in general COVID-19 patients (58) such as anorexia (2/27, 7%), nausea (2/27, 7%), and vomiting (2/27, 7%) are also present in COVID-19 KT patients. It should be noted that (as shown in case No. 2) the initial symptoms are fever and vomiting, which leads to a presumption of viral gastroenteritis. In this case, cough did not develop until 5 days later (43).

**Table 1:** Clinical characteristics, symptoms, and outcomes of 27 COVID-19 KT patients.

**No**.	**Age/gender**	**Years post-Tx**	**Symptoms**	**Mechanical ventilation**	**Prognosis**	**References**
1	52/M	12	Dyspnea, fever, cough, nausea, fatigue	No	Recover	(44)
2	50/M	4	Vomit, fever, cough	Intubation	Unkn	(43)
3	75/M	10	Cough, myalgia, fever, dyspnea	NIV	Death	(45)
4	52/F	0.67	Cough, myalgia, fever, dyspnea	NIV	Unkn	
5	38/M	0.25	Fever, cough	No	Recover	(46)
6	64/M	4	Fever, cough, sputum production, myalgia or fatigue	No	Unkn	
7	37/F	0.5	Fever, cough	No	Recover	
8	47/M	1	Fever, cough, sputum production, myalgia or fatigue	No	Unkn	
9	38/M	2.6	Fever, cough, sputum production, myalgia or fatigue	No	Recover	
10	49/M	6	Anorexia, fever, cough	No	Recover	(47)
11	58/M	12	Fever, cough, dyspnea	ECMO	Death	(48)
12	50/M	4	Fever, cough	No	Recover	(49)
13	49/M	2	Fever and respiratory symptoms	No	Recover	(50)
14	29/M	1	Fever, fatigue, chill, dyspnea, nasal stuffiness, anorexia, nausea and vomiting	No	Recover	(51)
15	36/F	18	Cough, fatigue, coryza	No	Recover	(52)
16	32/M	3	Fever, dyspnea, cough	No	Recover	(53)
17	58/M	3	Fever, dyspnea, cough	No	Unkn	(54)
18	48/M	17	Fever, cough, sputum production, myalgia, fatigue, chest tightness	No	Recover	(55)
19	28/F	0.5	Fever, malaise, sore throat, rhinorrhea	No	Recover	(56)
20	78/M	8.3	Fever, dyspnea	No	Death	(57)
21	73/M	1.8	Fever, dyspnea, cough	No	Unkn	
22	80/M	3.8	Dyspnea, cough, myalgia, hypoxia	No	Unkn	
23	71/F	6	Fever, dyspnea, cough, sore throat	NIV	Death	
24	71/M	30.1	Fever, epigastric pain	No	Unkn	
25	76/M	14.8	Fever, rhinorrhea	NIV	Recover	
26	39/M	16.8	Fever, myalgia	No	Unkn	
27	65/M	6.5	Fever, dyspnea, cough	NIV	Unkn	

*ECMO, extracorporeal membrane oxygenation; F, female; M, male; NIV, non-invasive ventilation; Unkn, unknown*.

Of the 27 COVID-19 KT patients, 7 (25.9%) required mechanical ventilation (Table 1). Based on the severity, this indicates that at least 25.9% of the COVID-19 KT patients were critical (defined as COVID-19 positive along with mechanical ventilation or evidence of multi-organ failure or shock) (59). In comparison, the condition of about 5% of COVID-19 patients in the general population are critical (60), which implies a worse prognosis among infected SOT recipients. The mortality rate was 30.8% (4/13) for all known outcomes (Table 1). This is consistent with a recent single-center study with a mortality rate of 28% (10/36) (61).

Antiviral treatment remains controversial since sufficient evidence is not available to prove its efficacy against SARS-CoV-2 (62, 63). The current proposed antiviral drugs with small volume randomized controlled trials include antimalarial drugs (chloroquine and hydroxychloroquine), HIV protease inhibitors (lopinavir/ritonavir, darunavir/cobicistat, darunavir/ritonavir), and remdesivir (64). On May 1, 2020, US FDA (Food and Drug Administration) issued an emergency use authorization for remdesivir for treatment of severe COVID-19 patients (65, 66). Of the 27 COVID-19 KT patients, 12 (44.4%) were given hydroxychloroquine, while 10 were prescribed lopinavir/ritonavir (Table 2). Patient No. 12 recovered with unaltered immunosuppressive therapy and no antiviral drug. Antiviral drugs must be handled with caution due to potential drug–drug interactions and adverse effects. The elimination of calcineurin inhibitors (CNI) and rapamycin inhibitors (mTORi) is mainly controlled by cytochrome P450 3A4 (CYP3A4), CYP3A5, and the efflux pump P-glycoprotein (67). However, both chloroquine and hydroxychloroquine are P-glycoprotein inhibitors and lopinavir/ritonavir are potent CYP3A4 inhibitors (68). In patient No. 15, tacrolimus trough level reached 90.5 ng/mL with a combination of lopinavir/ritonavir, hydroxychloroquine, and tacrolimus. The high tacrolimus level induced renal failure in patient No. 27. Transitional discontinuation and dose reduction of CNI are necessary before the initiation of the aforementioned antiviral drugs.

**Table 2:** Immunosuppressive agents changes, treatment, and allograft function of 27 COVID-19 KT patients.

**No**.	**CNI**	**mTORi**	**MMF/EC-MPS**	**Oral steroids**	**MEP pulse**	**Anti-viral therapy**	**Baseline creatinine**	**Creatinine on admission**	**Creatinine at discharge or last known creatinine**	**Proteinuria**	**Notes**	**References**
1	S	–	S	S	Y	Arbidol	139	143	104	Yes	Proteinuria disappear after recovery	(44)
2	S	S	–	C	N	HCQ + LPV/r	114.9	141.4	265	Unkn	Atypical initial symptoms: malaise, fever, vomiting without respiratory symptom	(43)
3	S	–	S	C	N	HCQ + LPV/r	185.6	194.5	194.5	Unkn		(45)
4	S	–	S	C	N	HCQ + DRV/c	114.9	212.2	123.8	Unkn	AKI, Systemic inflammation	
5	R	–	S	C	N	Oseltamivir or arbidol	Normal	98	Comparable^a^	Yes	Proteinuria disappear after recovery	(46)
6	C	–	S	S	N	Oseltamivir or arbidol	Normal	411.7	Decreased^b^	Yes	Methylprednisolone pulse therapy for rejection just before COVID-19 confirmed. Fever and dyspnea not improved after 28 days since confirmed	
7	S	–	S	C	N	Oseltamivir or Arbidol	Normal	137	Comparable	Yes	Proteinuria disappear after recovery	
8	S	–	S	C	N	Oseltamivir or Arbidol	Normal	146.9	Comparable	Yes		
9	C	–	C	C	N	Oseltamivir or Arbidol	Normal	135.4	Comparable	No		
10	S	–	S	S	Y	Arbidol + Ribavirin	110–120	167.3	119.2	No		(47)
11	–	–	S	S	Y	Oseltamivir	Normal	Unkn	Unkn	Unkn	Multiorgan failure (lung, kidney, and heart)	(48)
12	C	–	C	–	N	None	Unkn	150.3	121.1	Unkn		(49)
13	C	–	C	C	Y	LPV/r + Ribavirin	Unkn	128.1	101	Unkn		(50)
14	C	–	C	C	N	LPV/r	Unkn	102	113.8	Unkn	NAT test negative on illness day 3 but turned positive 2 days later	(51)
15	R	–	–	I	N	HCQ + LPV/r changed to DRV/c 2 days later	132.6	202.4	154.7	Unkn	Tac trough level reached 90.5 ng/ml due to drug-drug interaction	(52)
16	C	–	C	I	N	HCQ + Oseltamivir	168	229.8	247.5	Yes		(53)
17	–	–	S	–	N	None	140	133	111	Yes	Belatacept discontinued. NAT test still positive 23 days after COVID-19 confirmed	(54)
18	R	S	–	–	Y	Oseltamivir + Arbidol	Unkn	138	Comparable	Unkn		(55)
19	C	–	–	C	N	Oseltamivir	Unkn	81	Unkn	Unkn	Strict isolation at home with 2 clinic visits and one time 24-h hospitalization	(56)
20	R	–	–	C	N	LPV/r	Unkn	Unkn	Unkn	Unkn		(57)
21	R	–	S	S	N	LPV/r + HCQ	Unkn	Unkn	Unkn	Unkn	LPV/r discontinued due to high Tac level	
22	R	–	S	C	N	LPV/r + HCQ	Unkn	Unkn	Unkn	Unkn		
23	R	–	S	S	Y	LPV/r + HCQ	Unkn	Unkn	Unkn	Unkn		
24	R	–	–	–	N	HCQ	Unkn	Unkn	Unkn	Unkn		
25	–	C	S	C	Y	HCQ	Unkn	Unkn	Unkn	Unkn		
26	S	S	–	C	Y	HCQ	Unkn	Unkn	Unkn	Unkn	Readmission due to clinical worsening	
27	R	–	R	C	N	LPV/r + HCQ	Unkn	Unkn	Unkn	Unkn	Renal failure due to high Tac level induced by LPV/r	

*C, continue; CNI, calcineurin inhibitor; DRV/c, darunavir/cobicistat; HCQ, hydroxychloroquine; LPV/r, lopinavir/ritonavir; MEP, methylprednisolone; mTORi, mammalian target of rapamycin inhibitors; MMF, mycophenolate mofetil; EC-MPS, enteric-coated mycophenolate sodium; R, reduce; S, stop; Unkn, unknown*.

a *Comparable to serum creatinine at admission*.

b *Decrease after methylprednisolone pulse therapy*.

*In vitro* study shows that cyclosporin A inhibits diverse coronavirus replication including severe acute respiratory syndrome coronavirus (SARS-CoV) and middle east respiratory syndrome coronavirus (MERS-CoV) (69, 70). However, further *in vivo* and clinical studies are needed to support the conversion from tacrolimus to cyclosporine in COVID-19 SOT patients. Since mTOR inhibitors can cause interstitial pneumonitis and lead to ground glass opacity abnormality in chest CT scan (71), switching mTOR inhibitor to CNI should be considered among COVID-19 SOT patients with typical ground opacity chest CT scan.

The lack of an effective treatment strategy against COVID-19 implies that the recovery depends primarily on our immune system itself. Regular immunosuppression in SOT patients leads to a longer viral shedding time as observed from past experience with RNA respiratory viral infections (42). Although further evidence is needed, reduction of immunosuppression might contribute to a better prognosis and a shorter course of the disease. Of the 27 COVID-19 KT patients, 21 (77.8%) reduced or stopped their immunosuppressive agents (Table 2). CNI was reduced in 9/24 (37.5%) and stopped in 8/24 (33.3%) patients. Mycophenolate mofetil (MMF) or enteric-coated mycophenolate sodium (EC-MPS) was stopped in 14/20 (70%) patients and reduced in 1/20 (5%) patients. However, it is riskier to reduce immunosuppression in COVID-19 SOT cases than in SOT patients with opportunistic infections because immunocompetent people are generally susceptible to COVID-19 and it does not imply deficient immune status. It should be noted that 6 of the 7 (85.7%) COVID-19 KT patients with proteinuria were treated with reduced immunosuppression. Certainly, proteinuria could be caused directly by COVID-19 (72, 73), but it could also be an early sign of renal rejection (74). Further, the disappearance of proteinuria after COVID-19 recovery in patient Nos. 1, 5, and 7 might be related to the resumption of immunosuppression or remission from potential COVID-19 kidney impairment.

KT recipients are deeply concerned with changes in renal function. However, a recent postmortem renal histopathology study in COVID-19 patients showed the direct renal impairment caused by SARS-CoV-2, such as diffuse brush border damage of proximal tubule, vacuolar degeneration, and necrosis (75). Acute kidney injury (AKI) occurrence in COVID-19 patients varies drastically from 0 to 28% [0/116, 0% (76); 55/287, 19.2% (77); 523/2,634, 19.9% (78); 55/193, 28% (72)]. This variability may be related to the differences in therapy and treatment availability. Proteinuria is found in 28.57–59% of COVID-19 patients and hematuria is seen in 41.17–44% of COVID-19 patients (72, 73). This impairment of the kidney may be caused by hypoxemia, hemodynamic changes, and most importantly by circulating inflammatory mediators (79). During our review, we paid particular attention to kidney allograft function alteration. Only 1 of the 27 (3.7%) COVID-19 KT patients were mentioned with AKI. Besides proteinuria (7/9, 77.8%), no abnormal serum creatinine change was observed between creatinine level at admission and at discharge (or last follow-up).

Eight of the 27 (29.6%) COVID-19 KT patients were treated with methylprednisolone pulse therapy. Altogether, 81.5% (22/27) of the patients were prescribed with steroids including oral steroids. Theoretically, corticosteroid administration in COVID-19 KT patients could be beneficial in two ways: (1) immunosuppression to prevent allograft rejection and (2) alleviation of immune-related injury to the lung (80) or kidney (79). However, a retrospective cohort study showed that high-dose corticosteroids had a limited beneficial effect on SARS because it increased the risks of virus dissemination, opportunistic infection, and osteopathy (81). In patient No. 6, methylprednisolone pulse therapy (1,000 mg) was administrated to treat acute rejection just 1 day before the confirmation of COVID-19. A large dose of steroids could be an important cause of constant fever and dyspnea during hospitalization (28 days since the confirmation of COVID-19). Corticosteroid therapy has been a long-lasting controversy in viral pneumonias caused by coronaviruses. Its use in the critically ill subgroup may help improve the outcome (80); however, further studies are essential to establish guidelines for steroid therapy in COVID-19 SOT patients.

In short, compared with general population current reports on COVID-19 KT patients showed similar symptoms and implied worse outcomes. Further solid evidences are needed to prove the efficacy of immunosuppression reduction strategy, current antiviral treatment, and methylprednisolone pulse therapy. Despite of involvement of kidney in COVID-19 KT patients, no permanent renal function impairment was observed.

### COVID-19 in Liver Transplant Patients

Regarding COVID-19 infection in liver transplantation, a 37-year-old man developed fever with abdominal discomfort at day 9 (D9) after liver transplant and was subsequently confirmed as COVID-19 positive by RT-PCR and CT examination. The patients' immunosuppression was discontinued and he was administered low-dose methylprednisolone for 2 weeks. His temperature recovered normally at D18 post-transplant; however, he developed rejection at D27 post-transplant, which was eventually controlled using tacrolimus and an infusion of methylprednisolone for 3 days. His RT-PCR test for COVID-19 was negative at D45 post-transplant and he was discharged after achieving clinical cure standards at D51 post-transplant (55). Another 37-year-old man presented with fever (with a temperature of up to 39°C) 4 days prior to liver transplant, which persisted for 30 days even after antimicrobial agents and caspofungin were prescribed. Thoracic CT and RT-PCT test confirmed COVID-19 infection at D15 post-transplant that was eventually cleared at D49 post-transplant. However, acute cellular rejection was suspected due to low-dose immunosuppression at D36 post-transplant and the patient did not recover during hospitalization (82). Unfortunately, this patient did not undergo COVID-19 associated examinations before liver transplant; therefore, it is not known whether COVID-19 infection occurred before or after the transplant. A 50-year-old man who had a cadaveric liver transplant in July 2017 contracted COVID-19 and presented with a representative clinical course of the disease, including persistent fever, significant lymphocytopenia, and mixed diffuse ground-glass opacities on CT. This patient had no rejection phase and successfully recovered after 1 month of comprehensive treatment including immunosuppression adjustment, oxygen therapy, antivirus, systemic low-dose methylprednisolone, intravenous immunoglobulin, prophylactic antibiotic, alpha interferon, and nutritional support (83).

Unfortunately, several cases with fatal outcomes have also been reported. A 59-year old patient who had undergone a liver transplant 3 years earlier was infected by his wife on February 1, 2020. His symptoms progressed rapidly from mild to critical illness with several nosocomial infections, and although several standard rescue efforts were attempted, he eventually died 45 days after the diagnosis. The authors believe that an earlier withdrawal of immunosuppression along with a more aggressive approach that includes antiviral and antibacterial therapy might have averted the fatal outcome (84). A transplant center from Italy reported that three long-term (more than 10 years) liver transplant survivors died between 3 and 12 days after the onset of COVID-19 pneumonia. All three patients were men and older than 65 years with diabetes. The authors declare that long-term liver transplant patients with metabolic comorbidities are associated with a poor prognosis (85).

Similar with the difficulty we face in COVID-19 KT patients, aforementioned COVID-19 liver transplant cases show that it is tricky to regulate immune system and balance between infection and rejection. The liver tissue damage might be due to an immune-mediated inflammatory response to the virus or a direct virus-induced cytopathogenic effect that has been reviewed elsewhere (86).

### COVID-19 in Heart and Lung Transplant Patients

Compared with the COVID-19 infection in kidney and liver transplant patients, there are fewer reported cases for heart transplant patients. The first two cases of COVID-19 infected heart transplant recipients from China were cured after treatments that were similar to those in non-transplant recipients (87). Two of the three heart transplant recipients reported from Spain were discharged after 20 days of treatment, which included hydroxychloroquine (HCQ) therapy and discontinuation of immunosuppression; however, the third one died at day 10 after confirmed COVID-19 infection although lopinavir/ritonavir, HCQ, and interferon–β (IFN-β) were administered (57).

Lung transplantation is the only effective treatment modality for end-stage pulmonary chronic diseases (88). A 59-year-old woman 13 months post-bilateral lung transplant was reported with confirmed COVID-19. Only mild exercise dyspnea and dry cough were present without any fever or diarrhea. Without any administration of antiviral drugs or change of immunosuppression therapy or mechanical ventilation, the viral load decreased overtime and turned negative after 21 days hospitalization (89). Currently, no guidelines are available to treat lung transplant patients with COVID-19 pneumonia.

Lung transplant as a treatment of acute infectious pneumonia was relatively rare (90). During this COVID-19 pandemic, three chronic pulmonary disease patients infected with COVID-19 (for more than a month) received lung transplants after full ethical approval. Two of them survived during the short-term follow-up; however, the third patient died post-transplant (91). Soon afterwards, the first description of lung transplantation for COVID-19 confirmed elderly patients with end-stage pulmonary disease was reported by Han et al. (90). It is recommended that the RT-PCR test for COVID-19 should be negative at least twice (24-h interval) for transplant candidates; else, the virus may damage the transplanted lung and medical staff will be at a high risk of transmission. These five reported cases suggest that lung transplant is a potential option for patients when internal medicine treatments including mechanical ventilation and extracorporeal membrane oxygenation (ECMO) cannot enhance lung function.

## Conclusion

The COVID-19 pandemic will continue to threaten global public health and transplantation programs until effective antiviral drugs and vaccines are developed. In order to ensure transplantation safety, donor and recipient screening is necessary in COVID-19 spreading areas. The SOT recipients are an immunologically vulnerable group and it is crucial to prevent viral infection in such patients. Both online education to spread awareness about COVID-19 and online follow-up could minimize the risk of infection in SOT patients. Compared with the general population, our review of current COVID-19 KT cases show a similar clinical manifestation and inferior outcomes. The long-lasting controversy over immunoregulation to balance infection and rejection continues during this battle with COVID-19. Antiviral drugs and methylprednisolone pulse therapy should be administered with great caution. Current antiviral therapy needs more evidence to prove that its efficacy can outweigh its potential adverse effects and drug-drug interactions. Lung transplant could be a possible way out for end stage COVID-19 patients, but great caution should be taken before COVID-19 lung injury is proven irreversible. Generally, much more studies and specific clinical guidelines are needed to protect transplantation from the adverse effect of COVID-19.

## Author Contributions

HZ and HD wrote the manuscript. HZ generated the tables. HZ, HD, and XX revised the manuscript. All authors have contributed to the editing of the manuscript.

## Conflict of Interest

The authors declare that the research was conducted in the absence of any commercial or financial relationships that could be construed as a potential conflict of interest.
